# Differential Role of PKC-Induced c-Jun in HTLV-1 LTR Activation by 12-O-Tetradecanoylphorbol-13-acetate in Different Human T-cell Lines

**DOI:** 10.1371/journal.pone.0029934

**Published:** 2012-01-27

**Authors:** Ammar Abou-Kandil, Rachel Chamias, Mahmoud Huleihel, W. T. Godbey, Mordechai Aboud

**Affiliations:** 1 Shraga Segal Department of Microbiology and Immunology, Faculty of Health Sciences, Ben Gurion University of the Negev, Beer Sheva, Israel; 2 Department of Virology and Developmental Genetics, Faculty of Health Sciences, Ben Gurion University of the Negev, Beer Sheva, Israel; 3 Laboratory for Gene Therapy and Cellular Engineering, Department of Chemical and Biomolecular Engineering, Tulane University, New Orleans, Louisiana, United States of America; George Mason University, United States of America

## Abstract

We have previously shown that TPA activates HTLV-1 LTR in Jurkat T-cells by inducing the binding of Sp1-p53 complex to the Sp1 site residing within the Ets responsive region 1 (ERR-1) of the LTR and that this activation is inhibited by PKCalpha and PKCepsilon. However, in H9 T-cells TPA has been noted to activate the LTR in two consecutive stages. The first stage is activation is mediated by PKCetta and requires the three 21 bp TRE repeats. The second activation mode resembles that of Jurkat cells, except that it is inhibited by PKCdelta. The present study revealed that the first LTR activation in H9 cells resulted from PKCetta-induced elevation of non-phosphorylated c-Jun which bound to the AP-1 site residing within each TRE. In contrast, this TRE-dependent activation did not occur in Jurkat cells, since there was no elevation of non-phosphorylated c-Jun in these cells. However, we found that PKCalpha and PKCepsilon, in Jurkat cells, and PKCetta and PKCdelta, in H9 cells, increased the level of phosphorylated c-Jun that interacted with the Sp1-p53 complex. This interaction prevented the Sp1-p53 binding to ERR-1 and blocked, thereby, the ERR-1-mediated LTR activation. Therefore, this PKC-inhibited LTR activation started in both cell types after depletion of the relevant PKCs by their downregulation. In view of these variable activating mechanisms we assume that there might be additional undiscovered yet modes of HTLV-1 LTR activation which vary in different cell types. Moreover, in line with this presumption we speculate that in HTLV-1 carriers the LTR of the latent provirus may also be reactivated by different mechanisms that vary between its different host T-lymphocyte subclones. Since this reactivation may initiate the ATL process, understanding of these mechanisms is essential for establishing strategies to block the possibility of reactivating the latent virus as preventive means for ATL development in carriers.

## Introduction

Adult T-cell leukemia (ATL) is etiologically associated with human T-cell leukemia virus type 1 (HTLV-1) infection [1], [2]. Accumulating data indicate that the HTLV-1 bZipper protein (HBZ), originally discovered by Gaudray et al [3], plays an important role in the ATL pathology [4]–[7]. Other studies have attributed a similar importance for the ATL pathology to the HTLV-1- induced modulation of cellular microRNAs (miRNA) expression [8]–[11]. However, the multifunctional viral Tax oncoprotein is widely regarded as the critical factor for initiating the leukemic process leading to this malignancy. This role of Tax is linked mainly to its abilities to activate constitutive expression of major regulatory factors like the NF-κB [12]–[17] and to impair the cellular genome stability, which are reflected by enhanced DNA-mutagenesis and chromosomal aberrations, including chromosomal aneuploidy, on one hand [14], [18]–[22] and protecting the cells from the DNA damage-induced apoptosis on the other hand [14], [20]–[25]. In addition, a recent study has demonstrated that Tax induces reactive oxygen species (ROS) in a way that correlates with DNA damage and expression of cellular senescence markers, but not with apoptosis [26]. Since similar correlation of ROS induction with genomic instability, cellular senescence and tumorigenesis has been reported for several oncogenes like Myc [27], [28], Ras [29] and the EBV nuclear antigen-1 [30], it has been suggested that this pathway might be involved also in the HTLV-1leukemogensis.

Notably, shortly after infection the virus enters into a latent state [14], [18], [19], [31] during which Tax level in the carriers' infected T-lymphocytes is very low due to suppression of the viral gene expression [14], [31]. However, despite this low virus expression, substantial levels of specific antibodies and cytotoxic T-lymphocytes (CTLs) against Tax and other HTLV-1 antigenic epitopes can be detected in these carriers [14], [32]–[35]. Accumulating data indicate that these two arms of the anti HTLV-1 immune response play critical roles in suppressing the viral gene expression and conferring, thereby, its latency [14], [32]–[34], [36]–[39].

The low Tax level is presumably insufficient for exerting its complex oncogenic effects [14], [31]. Therefore, only a small minority (5–10%) of these carriers eventually develop ATL after long latency of 20–60 years. On this ground we hypothesize that the transition from latency to the leukemic progression occurs in these particular carriers due to reactivation of the latent virus, which consequently elevates Tax level to its oncogenic threshold. Moreover, since the initial Tax level in the virus-harboring cells is very low, it is reasonable to assume that this reactivation initiates by a Tax-independent mechanism. Furthermore, since the ATL cells contain no or very low Tax level [14], [19], [40] we assume that this reactivation is likely temporal. We speculate that the activated virus returns, after a while, back to latency due to re-mounting of the host anti HTLV-1 immune surveillance mentioned above. This presumption implies that the transiently elevated Tax may initiate the leukemic process, in a hit-and-hide manner. We postulate that during the temporal time of the virus activation, Tax may initiate the leukemic process by enhancing mutagenesis and other chromosomal aberrations in its harboring cells while protecting them from apoptosis induction [14], [20], [41], [42]. In this manner, some of these cells may acquire certain specific mutations that render them permanently resistant to apoptosis. Such cells can then further progress toward ATL by a stepwise accumulation of additional mutations even after re-silencing the activated virus and shutting off its Tax protein. This model complies with the low ATL incidence and the long time required for its development.

ATL is an aggressive lethal malignancy [1], [2] that does not respond to presently available anti cancer medications [7], [43]. Therefore, in addition to the current intense effort focused in many laboratories on developing new effective curing therapies for ATL [43]–[45], we suppose that it would be highly important to concentrate effort also on establishing strategies for blocking the possibility for developing this malignancy in healthy carriers. Of interest, in this context, is the study of Afonso et al [46] which has demonstrated in series of baboons naturally infected with simian T-lymphotropic virus type 1 (STLV-1), that treatment with a combination of valproate (an inhibitor of histone deacetylases) and azidothymidine (an inhibitor of reverse transcriptase) efficiently decreases their proviral load. Based on this model, the authors suggest that such treatments may be useful to reduce the risk of HAM/TSP in healthy HTLV-1 carriers with high proviral load. However, in view of the putative model of ATL development described above, it seems that avoiding potential re-activation of the dormant virus might be an ideal approach for achieving this aim in HTLV-1 carriers. A support to this idea has emerged from a recent study of Ratner et al [40] which indicates that the inefficiency of the chemotherapy treatment of ATL results from re-activation of the dormant virus harbored in the leukemic cells by the DNA damage- and stress-inducing effects of the chemotherapeutic drugs. Taken together, blocking the potential re-activation of the dormant virus might both, prevent ATL development in healthy HTLV-1 carriers and improve the outcomes of the chemotherapy treatment in ATL patients. Establishing such an approach requires a deep understanding of the various optional complex mechanisms and factors that might potentially induce this viral reactivation.

In pursuing this issue, we have previously noted that HTLV-1 LTR expression can be activated in various human T-cell lines and primary T-lymphocytes by DNA-damaging and other stressing-inducing agents in absence of Tax [41], [47]. These findings suggest that environmental or intrinsic stress-inducing factors might trigger the viral re-activation in latent carriers by similarly activating its LTR. It should be pointed, however, that all the LTR-activating agents that we have tested can also induce apoptosis [41]. Moreover we have proved that this LTR activation depends on certain factor(s) participating in the apoptotic cascade by showing that it can be abrogated by the anti apoptosis factor Bcl-2 [41]. It might have seemed paradoxical to expect that reactivation of the virus by a mechanism that sets the host cells of the activated virus into apoptotic death, can lead to ATL. However, this apparent paradox has been reconciled by our observation that HTLV-1 infected T-cells, which display active viral gene expression, are protected by the viral Tax protein from apoptosis induction by such stress agents [41]. This finding implies that the Tax protein, emerging after re-activation of the dormant virus, can rescue its host cells from apoptosis and enable their leukemogenic progression.

In the last few years we have focused mainly on the activation of HTLV-1 LTR by 12-*O*-tetradecanoylphorbol-13-acetate (TPA) [41], [47]–[49]. This phorbol ester is a potent PKC activator [50] which can induce DNA damage by generating reactive oxygen species (ROS) [51]–[53]. Therefore, it can serve as an experimental model for revealing possible mechanisms of re-activation of the dormant virus in HTLV-1 carriers by certain PKC-activating physiological and pathological processes involving ROS production [54]–[57]. Our recent study [47] has shown that, although TPA activates the same PKC isoforms (α, β1, β2, δ, ε, and η) in both Jurkat and H9 human T-cell lines, these cells vary from each other in the rate of the ubiquitin-mediated downregulation of these PKCs [58] and in the effect of each individual PKC on the LTR expression. Moreover, these differences in the PKC characteristics have been proved to confer two different cell-type-dependent modes of LTR activation by TPA in these two cell lines. In Jurkat cells this activation is exerted via binding of an Sp1-p53 complex to Sp1 site residing within the 44 bp Ets responsive region-1 (ERR-1) located between the nucleotides −160 and −116 away from the transcription starting point of the LTR [47], [49]. This binding is inhibited by PKCα and PKCε. Therefore, the onset of the LTR activation occurs in these cells only after 12–24 hr of TPA treatment, i.e. the time that these two PKCs are depleted from these cells.

By contrast, in H9 cells TPA has been noted to activate the LTR in two consecutive phases. The first phase starts shortly after exposing the cells to TPA and peaks after 36–48 hr of TPA treatment. This activation depends on PKCη activity which is depleted from these cells only after 48–60 hr of TPA treatment and it critically requires the involvement of all the three repeated 21 bp Tax responsive elements (TRE) of the LTR [47]. The second phase starts after 72 hr of TPA treatment. Its mechanism is analogous to that of Jurkat cells, except that it is inhibited by PKCδ, which is also depleted from H9 cells only after 48–60 hr of TPA treatment [47]. Interestingly, the potent PKC inhibitor, bisindolylmaleimide-I (BI) [59], inhibits all the above mentioned PKC isoforms in both cell types, except PKCδ, which is sensitive to this inhibitor in Jurkat but resistant to it in H9 cells [47]. Therefore, BI can abolish the PKCα and PKCε antagonism to the LTR activation in Jurkat cells but not the PKCδ antagonism in H9 cells [47].

The aim of the present study was to identify the downstream factors mediating the specific effects of these PKC isoforms on the LTR expression in these two T-cell lines. Our experiments revealed that the first LTR activation phase in H9 was mediated by non-phosphorylated c-Jun that was transiently elevated in these cells by PKCη and that this elevated form of c-Jun activated the LTR by its binding to the AP-1 site residing in each of its three 21 bp TRE repeats. No such TRE-dependent LTR activation was observed in Jurkat cells, since only phosphorylated c-Jun, which could not bind to the AP-1 site, was elevated in these cells by PKCα and PKCε. Phosphorylated c-Jun was transiently elevated in H9 cells too, but this elevation was mediated by PKCδ. In both cells the phosphorylated c-Jun physically interacted with the Sp1-p53 complex and inhibited, thereby, its binding to ERR-1. Consequently the onset of the ERR-1-dependent LTR activation occurred in both cell lines only after depletion of their phospho-c-Jun-elevating PKCs by their downregulation.

## Results

### Transient elevation of phosphorylated and non-phosphorylated c-Jun in TPA-treated H9 cells

**Figure 1:**
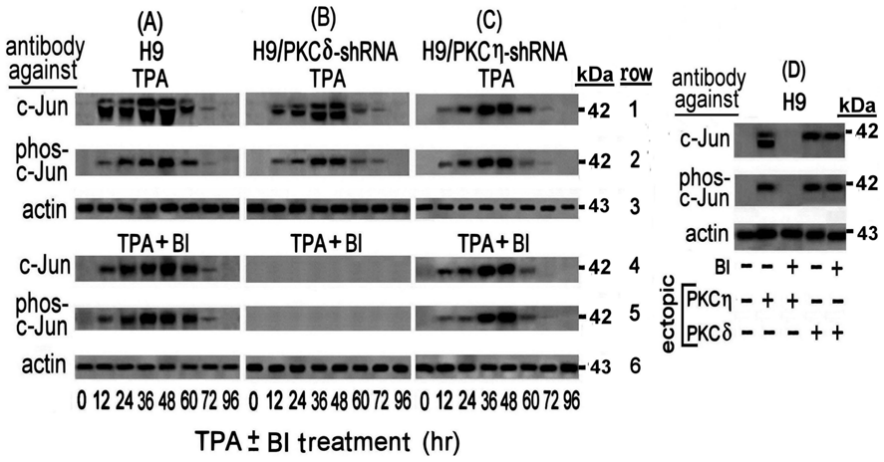
Transient Elevation of c-Jun and phospho-c-Jun by PKCη and PKCδ in TPA-treated H9 cells. H9 cells (A) and their subclones stably transfected with shRNAs against either PKCδ (B) or PKCη (C), were treated with TPA in absence (rows 1 to 3) or presence (rows 4 to 6) of the PKC inhibitor BI. Aliquots of the whole-cell extracts, prepared at the indicated times of the TPA ± BI treatment, were subjected to Western blot analysis with monoclonal antibody against c-Jun (rows 1 and 4). Then the blots were re-processed with anti phospho-c-Jun antibody (rows 2 and 5). Equal sample loading was assessed by re-processing the blotted filter with anti actin antibody (rows 3 and 6). (D) **Elevation of c-Jun and phospho-c-Jun by ectopically introduced PKCη and PKCδ**. H9 cells were transfected with plasmids expressing constitutively active PKCη or PKCδ in the absence or presence of BI. At 24 hr post transfection the cells were examined for the level of c-Jun and phospho-c-Jun by Western blot analysis as detailed for panels A, B and C. Non-transfected cells served as control.

We began this study by exploring the mechanism of the first phase of the LTR activation in the TPA-treated H9 cells. We have previously noted that this activation requires all the three 21 bp TRE repeats and that a single nucleotide substitution at the AP-1 binding sequence (see in “Materials and Methods”) of any of these TREs abolished this activation [47]. Furthermore, Jeang et al have shown that c-Jun activates the HTLV-1 LTR by binding to these AP-1 sites [62]. In addition, TPA-activated PKC isoforms have been reported to induce the expression of c-Jun and to affect its functions [65]–[67]. Collectively, these data prompted us to investigate whether TPA induced the first LTR activation phase by affecting c-Jun expression. Western blot analysis of c-Jun level in the whole cell extracts of TPA-treated H9 cells with anti c-Jun antibody revealed a transient increase of a massive band that peaked at 36–48 hr of the TPA treatment (Figure 1A row 1). However, in addition to this band, this antibody detected also an upper thinner band. To clarify the nature of these two bands the blotted filter was reprocessed with anti phospho-c-Jun antibody that specifically identifies c-Jun protein phosphorylated at serine residues 63 and 73. Figure 1A row 2, shows that this antibody identified only one band that was identical to the upper band noted in row 1 in terms of its electrophoretic migration and intensity. These results indicated that the upper band represented a c-Jun protein phosphorylated at serine residues 63 and 73, whereas the lower band, seen in row 1, evidently presented a c-Jun without this phosphorylation (that we referred to as c-Jun or non-phosphorylated c-Jun).

### Role of PKCη and PKCδ in the c-Jun/phospho c-Jun elevation

The decline of these two bands (Figure 1A, rows 1 and 2) coincided with the timing of the PKCη and PKCδ depletion from the TPA-treated H9 cells noted in our previous study [47]. In addition, we have previously found that PKCη suppresses the activity of PKCδ [47]. Therefore, it seemed reasonable to assume that PKCη alone was involved in the elevation of both c-Jun forms. However, this presumption was refuted by our next finding that the PKC inhibitor BI abolished only the lower band of the non-phosphorylated c-Jun, but not the upper band of the phospho-c-Jun that was detected by both, the anti c-Jun (Figure 1A row 4) and the anti phospho-c-Jun (Figure 1A row 5) antibodies. Of note in this context, we have previously shown also that in H9 cells BI inhibited the activity of PKCη but not of PKCδ [47]. On this ground the results presented in Figure 1 can be explained by postulating that in absence of BI the active PKCη suppressed the activity of PKCδ and therefore, only PKCη could evidently be involved in the elevation of both forms of c-Jun (Figure 1A, rows 1 and 2). On the other hand, blocking PKCη activity by BI most likely relieved the PKCη-mediated suppression of the BI-resistant PKCδ. Therefore, the single band remaining in these conditions, which was detected by both, the anti c-Jun (Figure 1A rows 4) and the anti phospho-c-Jun (Figure 1A rows 5) antibodies, was evidently of phospho-c-Jun that was elevated by the reactivated PKCδ. This presumption was supported by the following set of observations: (a) Knockdown of PKCδ with specific shRNA in absence of BI had no effect on the elevation of either of these two c-Jun forms (Figure 1B, rows 1 and 2). This finding confirmed that the active PKCη alone could indeed, elevate both of the c-Jun forms. (b) Knockdown of PKCδ in the presence of BI diminished the elevation of both c-Jun forms (Figure 1B rows 4 and 5). This finding confirmed that the BI-reactivated PKCδ was responsible for the phospho-c-Jun elevation in absence of PKCη which is shown in Figure 1A rows 4 and 5. (c) Knockdown of PKCη by shRNA led to elevation of only the phospho-c-Jun in both absence (Figure 1C rows 1 and 2) and presence (Figure 1C rows 4 and 5) of BI. This observation re-confirmed that the absence of PKCη activity permitted PKCδ to elevate phospho-c-Jun even in the presence of BI. (d) Transfecting TPA-non-treated H9 cells with plasmid expressing constitutively active PKCη resulted in elevation of the two c-Jun forms which were both abolished BI, whereas transfecting these cells with plasmid expressing constitutively active PKCδ elevated only the phospho-c-Jun and this elevation was unaffected by BI (Figure 1D).

Interestingly, the elevation time-course of the two c-Jun forms in the TPA-treated H9 cells (Figure 1A rows 1 and 2) closely correlated to the timing of first LTR activation phase that we previously noted in these cells [47]. However, while our previous study revealed that BI abolished the first LTR activation phase [47], the present experiment demonstrated that this inhibitor prevented only the elevation of the non-phosphorylated but not of the phosphorylated c-Jun (Figure 1 rows 4 and 5). These data suggest that the non-phosphorylated c-Jun, but not the phospho-c-Jun might be participating in this first LTR activation phase.

### Only the non-phosphorylated c-Jun binds to the 21 bp TRE repeats

**Figure 2:**
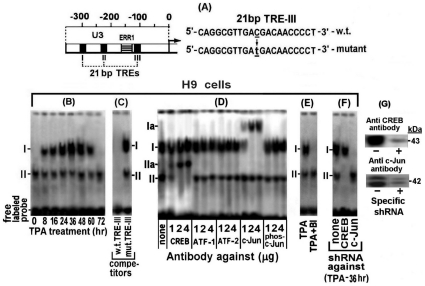
Binding of c-Jun and CREB of the TPA-treated H9 cells to TRE III. (A) Schematic illustration of the U3 region of the LTR. In addition, this scheme illustrates the employed probes carrying the wild type (w.t.) and the mutated TRE III sequences. (B) Aliquots of nuclear extracts prepared from H9 cells at the indicated times of the TPA treatment were analyzed by electrophoretic mobility shift assay (EMSA) for binding to 3′ biotin labeled oligonucleotide probe carrying the w.t. TRE III sequence. (C) The specificity of this binding was assessed with nuclear proteins of the cells prepared after treatment with TPA for 36 hr. This was done by competition with 50 fold molar excess of unlabeled oligonucleotides carrying the w.t (left lane) versus the mutant TRE III (right lane) sequences. (D) The nuclear proteins of the 36 hr TPA-treated H9 cells that bound to the TRE III probe were identified by supershift analysis with the indicated doses of antibodies against CREB, ATF-1, ATF-2, c-Jun and phospho-c-Jun. (E) BI effect on the nuclear protein binding to TRE III was determined by testing it the nuclear extracts of the H9 cells treated for 36 hr with TPA±BI. (F) H9 cells (control) and their sub-clones carrying either the anti CREB or anti c-Jun shRNA were treated with TPA for 36 hr. Then their nuclear extracts were subjected to EMSA with the labeled TRE III probe. (G) Illustration of the knockdown efficiency of CREB (upper blot) and phospho/none-phospho c-Jun by their specific shRNA (lower blot) in H9 cells treated with TPA for 36 hr.

Next, we explored why the non-phosphorylated rather than the phosphorylated-c-Jun was involved in this LTR activation and whether it exerted this effect directly or through an additional mediator. As mentioned before, Jeang et al [62] have reported that Jun activates the LTR by binding to its TRE sites. Based on this report, we elucidated whether the differential LTR activating potential of these two c-Jun forms, observed in our present experiment, reflected a variation in their TRE-binding capacity. This was done by examining the binding of the nuclear proteins of H9 cells at various time-points of their TPA treatment to the 3′-biotin-labeled TRE-III oligonucleotides probe shown in “Materials and Methods” and Figure 2A, which was arbitrarily chosen as a representative TRE. The EMSA results depicted in Figure 2B revealed two bands which were marked as I and II. The intensity of band I transiently increased, peaking at 36–48 hr of the TPA treatment and then declined and was undetected at 72 hr of the treatment. On the other hand, band II was not affected by this treatment.

The specificity of these bands was assessed by a competition with 50 fold molar excess of unlabelled w.t. 21 bp TRE-III oligonucleotide and its mutant illustrated in “Materials and Methods” and Figure 2A. This analysis was carried out with the nuclear extract of cells treated with TPA for 36 hr, which, as noted in our previous study [47], was the peak time-point of the first LTR activation phase. Figure 2C shows that the unlabeled w.t. TRE-III oligonucleotide completely abolished both bands whereas no competition was displayed by the mutant oligonucleotide.

The proteins forming these bands were identified by supper-shift analyses with the indicated antibodies. To ensure the reliability of this assay increasing doses of the tested antibodies were employed. Figure 2D revealed that low dose (1 µg) of antibody against the non-phosphorylated c-Jun supershifted only part of the TPA-responding band I to position Ia, whereas higher doses of this antibody quantitatively supershifted increasing amounts of this band, reaching a saturation at about 2 µg of the antibody. However, no supershift of this band was detected with the anti phospho-c-Jun antibody. These data indicated that only the non-phosphorylated c-Jun was involved in forming this band. On the other hand, band II was supershifted by anti CREB antibody to position IIa in a similar antibody-dose dependent manner. This band was not affected by anti c-Jun and anti phospho-c-Jun antibodies and conversely anti CREB antibody had no effect on band I (Figure 2D). These findings are in lne with the presumption that c-Jun and CREB cannot concomitantly bind to the same molecules of this probe, since their binding sites in the TREs share partially overlapping sequences [62]. Furthermore, consistent with this conclusion, Figure 2E shows that BI abolished the formation of band I by the PKCη-induced non-phosphorylated c-Jun, but not of the PKC-independent formation of band II by CREB. In addition, this BI-mediated inhibition of band I formation indicated that phospho-c-Jun, which was elevated in H9 cells by the BI resistant PKCδ (see Figure 1C), could not bind to TRE. This finding re-confirmed that only the non-phosphorylated c-Jun was involved in band I formation.

The activating transcription factor (ATF) family includes a large group of leucine zipper (bZIP) transcription factors [68] that regulate the expression of many cellular and viral genes in response to a wide range of intrinsic or external signals [69]–[71]. Under certain circumstances members of this family form transcriptional regulatory complexes with CREB (CREB/ATF) that bind to CRE sites of various promoters [72]. CREB/ATF-1 and CREB/ATF-2 have been reported to bind to the CRE site located in each of the 3 TREs of the HTLV-1 LTR [63], [64], [73]. These reports prompted us to check whether ATF-1 and/or ATF-2 joined CREB in forming band II in our present experiments. However, this possibility was ruled out by the results illustrated in Figure 2 D which demonstrated that band II was not supershifted by anti ATF-1 or anti ATF-2 antibody. To further substantiate the notion that band I was formed only by the non-phosphorylated c-Jun and band II by CREB, we used H9 cells stably transfected with anti CREB or anti c-Jun shRNA. These cells were treated with TPA for 36 hr (the peak time-point of the first the LTR activation phase) and examined for CREB and c-Jun binding to the 3′ biotin labeled TRE III probe. Cells without shRNA served as control. Figure 2F shows that, while the extract of the control cells formed both bands I and II. On the other hand, the extract of the cells carrying the anti CREB shRNA did not form band II, whereas the extract of the cells carrying the anti c-Jun shRNA did not form band I.

**Figure 3:**
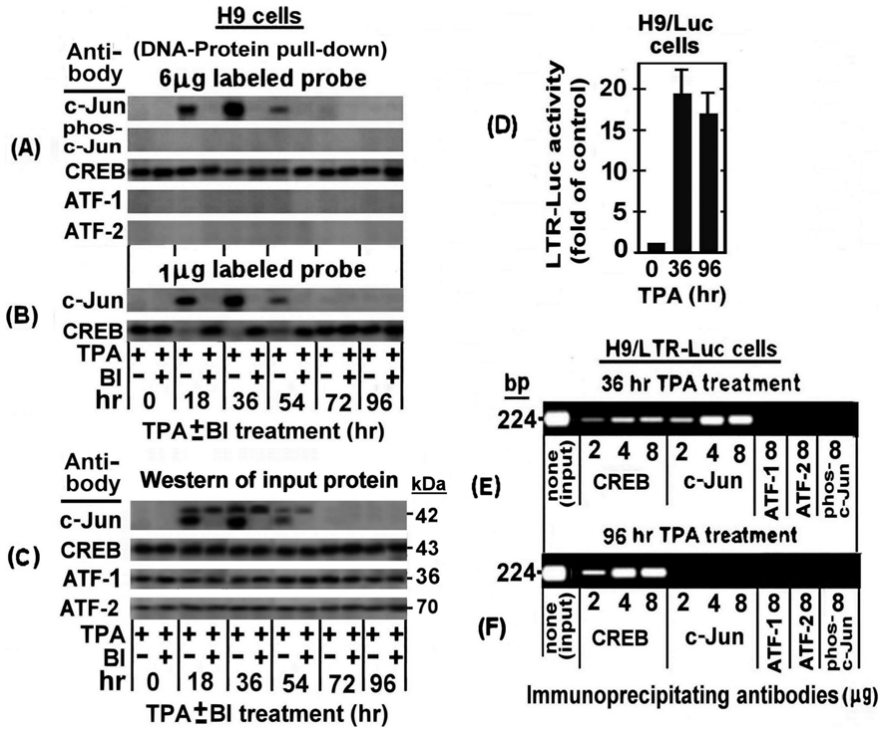
DNA-protein pull-down and Chromatin immunoprecipitation (ChIP) analyses of binding of nuclear proteins of TPA±BI treated H9 cells to TRE III. (A) For the DNA-protein pull-down analysis, nuclear extracts were prepared from H9 cells after treatment with TPA±BI for the indicated times. Aliquots (200 µg protein) of these extracts were reacted with 6 µg of 3′-biotin-labeled TRE III probe and further processed as detailed in “Materials and Methods”, using the indicated antibodies for identifying the specifically bound proteins. (B) The same analysis was performed as in panel A except that limiting dose (1 µg) of the 3′-biotin-labeled probe was employed in order to check for binding competition between c-Jun and CREB on the limited available AP1 site. (C) The same extracts were subjected to Western blot analysis for assessing the input of all the proteins tested in the experiment of panels A and B. To visualize an intracellular recruitment of nuclear proteins of H9 cells to TREs of a putative integrated HTLV-1 viral genome during the first and the second phases of the TPA-induced LTR activations we employed the ChIP procedure described in “Materials and Methods”. This was done with chromatins derived from H9 cells carrying stably transfected LTR-Luc plasmid (H9/LTR-Luc), that were exposed to TPA for 36 hr (the peak time-point of the first phase) or for 96 hr (the second phase). (D) These cells were assessed, first, for the activation of the integrated LTR-Luc in both time-points of the TPA treatment (right and middle panels). Untreated cells served as control (left column). The depicted results represent the average of the triplicate assays ± SE. Then the chromatin of the cells treated with TPA for 36 hr (panel E) and 96 hr (panel F) was subjected to ChiP analysis for binding of CREB, c-Jun, ATF-1, ATF-2 and phospho-c-Jun using the indicated doses of the corresponding precipitating antibodies.

Next, we analyzed the binding of nuclear proteins of the TPA ± BI treated H9 cells to the TRE III probe by the DNA-protein pull-down assay described in “Materials and Methods”. Figure 3A demonstrates that in absence of BI the amount of the non-phosphorylated c-Jun pulled-down by the anti c-Jun antibody, transiently increased, peaking at 36 hr of the TPA treatment and then depleted from the cells at 54–72 hr of the treatment. On the other, hand no pull-down of phospho-c-Jun was detected by the anti phospho-c-Jun antibody. In addition, this Figure shows that BI avoided the binding of the non-phosphorylated c-Jun to this probe. These data substantiate our earlier notions that band I is formed only by the non-phosphrylated c-Jun (see Figure 2D) and that BI avoids the formation of this band (see Figure 2E) by blocking the PKCη-induced elevation of the non-phosphrylated c-Jun (see Figure 1A row 5 and 1B row 4). The binding specificity of the nuclear proteins of these cells to TRE III was confirmed by our next finding that non-phosphorylated c-Jun was not pulled down in a similar assay using the mutated 3′-biotin labeled TRE III oligonucleotide (data not shown).

As noted above, CREB and c-Jun cannot bind jointly to the same individual TRE III probe molecule due to their partial overlapping binding site. This notion can readily be illustrated by EMSA since this assay reveals two separate bands (see Figure 2B and 2D) whose proteins can be identified by supershift with specific antibodies (see Figure 2D) and by distinguishing which of them is abolished by BI (see Figure 2E). However, it is rather more complicated to illustrate this exclusive binding to different individual molecules of the probe by the DNA-protein pull-down procedure. To overcome this obstacle we employed a small amount (1 µg) of the labeled probe, which was adjusted to be limiting factor that forced CREB and c-Jun to compete for its binding. The results depicted in Figure 3B show, indeed, that when c-Jun level was elevated by TPA it displayed an increasing binding to the probe that was paralleled by a reciprocal decline of the CREB binding, whereas this CREB binding to the probe was gradually restored along with the downregulation of the c-Jun level at longer TPA treatment.

As expected, CREB binding to this probe was not affected by TPA nor by BI (Figure 3A). This finding supported our conclusion from the data presented in Figure 2D that CREB was not involved in the LTR activation by TPA. Moreover, neither ATF-1 nor ATF-2, were found to bind to this probe although, as can be seen in Figure 3C, they were detected by the Western analysis of the input proteins tested in Figures 3A and 3B. This finding thus re-confirmed that ATF-1 and ATF-2 did not participate in the first phase of the TPA-induced LTR activation.

In the next experiment, we employed the ChIP assay for visualizing an in vivo recruitment of the nuclear factors to the TREs of integrated HTLV-1 DNA in the cellular genome. This was done with the H9 cells stably transfected with LTR-Luc (H9/LTR-Luc) which provided a convenient cellular system for this assay. These cells were treated with TPA for 36 hr (i.e. the peak time point of the c-Jun/phospho-c-Jun elevation) and 96 hr (i.e. when c-Jun and phospho-c-Jun were already depleted from the cells, see Figure 1A). We analyzed first the TPA activation of the integrated LTR-Luc. Figure 3D demonstrates that this reporter was substantially stimulated at both time-points of the TPA treatment. Then the fragmented chromatin of these cells was immunoprecipitated by increasing amounts of the indicated antibodies and the DNA was purified from the precipitated fragments and amplified by real-time PCR with primers flanking the region spanning the three TREs of the LTR. Figure 3E shows that at 36 hr of the TPA treatment these TRE-containing DNA fragments were precipitated by both the anti CREB and the anti c-Jun antibodies and that in both cases the amount of the precipitated fragments increased along with the increasing doses of the antibodies, reaching a saturation at 2 µg antibody. Notably at this time point of the TPA treatment the amount of the fragments precipitated with anti c-Jun antibody was considerably higher than that which was precipitated with the anti CREB antibody, thus confirming that the level of c-Jun was higher than that of CREB. On the other hand, Figure 3F shows that at 96 hr of the TPA treatment, i.e. when no c-Jun was left in the cells (see Figure 1A), chromatin precipitation was detected only with anti CREB antibody. Moreover, the amount of the fragments precipitated by this antibody was higher at the 96 hr than at the 36 hr time-point. This finding indicates that there was no c-Jun at the 96 hr time-point to compete with CREB for the TRE. In addition, no precipitation of these fragments was detected with antibodies against ATF-1, ATF-2 and phospho-c-Jun in both time-points, which further confirmed that these factors were not involved in the LTR activation by TPA.

### Direct confirmation that only the non-phosphorylated c-Jun mediates the first phase of the LTR activation in TPA-treated H9 cells

**Figure 4:**
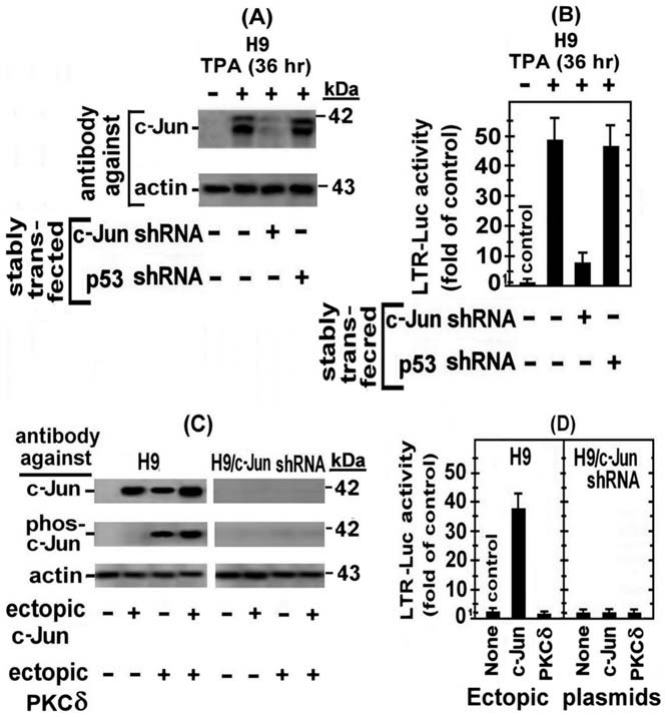
The first LTR activation phase in TPA-treated H9 cells is mediated by the non-phosphorylated c-Jun. H9 cells and their subclones stably transfected with c-Jun shRNA (H9/c-Jun shRNA) or with p53 shRNA (H9/p53 shRNA) were treated with TPA for 36 hr (the peak time-point of the first phase). (A) Whole cell extracts were prepared from part of the treated cells for measuring the level of the non-phosphorylated c-Jun (c-Jun) by Western blot analysis with anti c-Jun antibody. Equal sample loading was assessed by re-processing the blotted filter with anti actin antibody. TPA-untreated H9 cells without shRNA served as negative control, whereas TPA-treated H9 cells without shRNAs served as positive control. (B) The remaining cells were transfected with the LTR-Luc reporter. TPA-untreated cells without shRNAs served as negative control and TPA-treated cells without shRNAs served as positive control. The pRL-renilla plasmid was included in this and all the subsequent transient transfection experiments as internal control for assessing variation in the transfection efficiency. The enzymatic activities were measured at 24 hr post transfection and the Luc activity was normalized to that of renilla and plotted as fold of the respective control. The documented results presented the average of triplicate transfections ± SE. (C) H9 cells without (left panels) and with (right panels) anti c-Jun shRNA were transfected with plasmid expressing non-phosphorylated c-Jun (ectopic c-Jun) or with plasmid expressing constitutively active PKCδ which elevates only phospho-c-Jun. Cells without these ectopic plasmids served as control. At 24 hr after transfection the whole cell extracts of the transfected and non-transfect cells were subjected to Western blot analysis with anti-c-Jun antibodies (top panels) and with antibodies detecting only phosphorylated c-Jun (middle panels). Equal sample loading was assessed with anti actin antibody (bottom panels). (D) H9 cells (left panel) and their subclone stably transfected with anti c-Jun shRNA (H9/c-Jun shRNA, right panel) were transiently transfected with LTR-Luc alone (control) or together with ectopic c-Jun- or ectopic PKCδ- expressing plasmids. Calculation of the enzymatic activities and their presentation were as in Figure 4B.

It was important, at this stage, to assess the biological roles of the two c-Jun forms in the cellular mechanism of the first LTR activation phase in the TPA-treated H9 cells. For this purpose we explored, first, the effect of c-Jun knockdown on this activation by exposing H9 cells stably transfected with the plasmid expressing anti c-Jun shRNA (H9/c-Jun-shRNA) and their parental H9 cells to TPA for 36 hr, which was the peak time-point of the first LTR activation phase. As control for the specificity of this shRNA, we employed, in parallel, H9 cells stably transfected with an arbitrarily chosen irrelevant shRNA-expressing plasmid, like the anti p53 shRNA (H9/p53 shRNA). Part of these cells were used for measuring the level of c-Jun in their whole cell extracts by Western blot analysis, whereas the remaining cells were transfected with the LTR-Luc reporter and tested for its expression at 24 hr post-transfection. This experiment revealed that the anti c-Jun shRNA almost completely abolished the elevation of both c-Jun forms (Figure 4A, upper panel) and strongly reduced the LTR activation (Figure 4B), whereas the anti p53 shRNA, which strongly inhibited p53 formation (not shown), was ineffective in both parts of this experiment (Figures 4A and 4B). To find out which of the two c-Jun forms mediated this LTR activation, TPA-untreated H9 cells were transfected with LTR-Luc alone (control) or together with c-Jun-expressing plasmid that produced only non-phosphorylated c-Jun (see Figure 4C), or with plasmid expressing ectopic PKCδ which, as noted before, elevated only phospho-c-Jun (see Figure 1C). This experiment revealed that the ectopic non-phosphorylated c-Jun markedly stimulated the LTR-Luc expression in H9 cells, whereas the PKCδ-elevated phospho-c-Jun was slightly inhibitory (Figure 4D, left panel). As expected, neither the ectopic c-Jun nor the ectopic PKCδ had any effect on the LTR-Luc expression in H9/c-Jun-shRNA (Figure 4D, right panel).

### TPA induces in Jurkat cells a transient elevation of only phospho-c-Jun through a cooperative action of PKCα and PKCε

**Figure 5:**
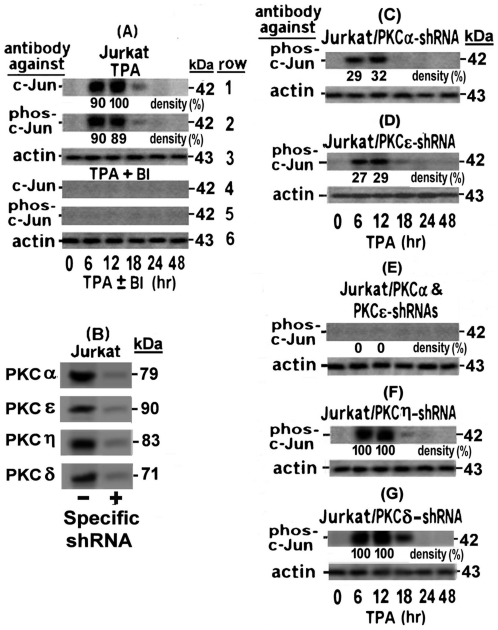
Role of PKCα and PKCε in the phospho-c-Jun elevation in TPA-treated Jurkat cells. (A) Jurkat cells were treated with TPA in absence (rows 1–3) or presence (rows 4–6) of the inhibitor BI. Aliquots of the whole-cell extracts, prepared from the cells at the indicated times of the TPA± BI treatments, were subjected to Western blot analysis, first with anti c-Jun, then with anti phos-c-Jun and finally with anti actin antibody as in Figure 1. Panel (B) shows the efficiency of the specific shRNA-mediated knockdown of the indicated PKCs isoforms to be employed in the experiments illustrated in the next panels. Jurkat subclones stably transfected with anti PKCα (B) or anti PKCε (C) shRNAs or with both of them (D) were treated with TPA± BI for the indicated times and then examined by Western blot for the level of phos-c-Jun and actin panel (A). As negative control, similar analyses were performed with Jurkat cells transfected with anti PKCη (F) or anti PKCδ (G) shRNAs. To quantify the effect of these knockdowns on the level of the tested c-Jun and phosho-c-Jun we performed densitometry measurements of the bands in the original exposed films of the Western blots. The results are presented as % of the largest band in Figure 1A row 1 which was designated as 100%. To assess the knockdown effects of the employed shRNAs on their target PKC isoforms (G), whole cell extracts of Jurkat cells without (left) or with (right) the specific shRNA against the indicated PKCs were subjected to Western blot analysis with the respective antibodies.

To explore why Jurkat cells do not display the TRE-mediated LTR activation by TPA [47] we investigated the effect of TPA on their c-Jun status. Western blot analysis of the whole-cell extracts of these cells revealed that anti c-Jun antibody detected a transient elevation of only one band (Figure 5 B, row 1) which was recognized also by the anti phospho-c-Jun antibody (Figure 5 B, row 2). This finding indicates that in Jurkat cells TPA induces an elevation of only the phospho-c-Jun. This elevation peaked at 6–12 hr of the TPA treatment. Its subsequent decline (Figure 5A, rows 1 and 2) coincided with the previously reported timing of PKCα and PKCε depletion from these cells [47]. Moreover, our present experiment demonstrated that this elevation was blocked by BI (Figure 5A, rows 4 and 5). Since both PKCα and PKCε have been previously proved to be sensitive to BI [47], these two observations can be interpreted as suggesting that the phospho-c-Jun elevation was mediated either by one of these PKCs or by a cooperative action of both of them. To distinguish between these possibilities we examined the elevation of phospho-c-Jun in TPA-treated Jurkat cells stably transfected with anti PKCα shRNA (Jurkat/PKCα-shRNA) or anti PKCε shRNA (Jurkat/PKCε-shRNA). Figure 5B shows that both PKCα and PKCε were strongly suppressed by their specific shRNAs. Nevertheless, although the phospho-c-Jun elevation was substantially reduced by this strong silencing of PKCα (Figure 5C) or PKCε Figure 5D), this inhibition was incomplete (compare the bands in these figures to those of Figure 5A rows 1 and 2). Only when both shRNAs were introduced (Jurkat/PKCα+PKCε), the elevation of phospho-c-Jun was almost completely omitted (Figure 5 E). These data indicated that both PKCs jointly participated in achieving the full extent of this elevation. Knockdown of PKCη (Figure 5F) and PKCδ (Figure 5G) by their specific shRNAs had no effect on the phospho-c-Jun elevation, thus confirming the specificity of the joint role of PKCα and PKCε in the phospho c-Jun elevation in the TPA-treated Jurkat cells.

### Jurkat cells display only CREB binding to TRE but can be rendered to display TRE-dependent LTR activation by ectopic c-Jun

**Figure 6:**
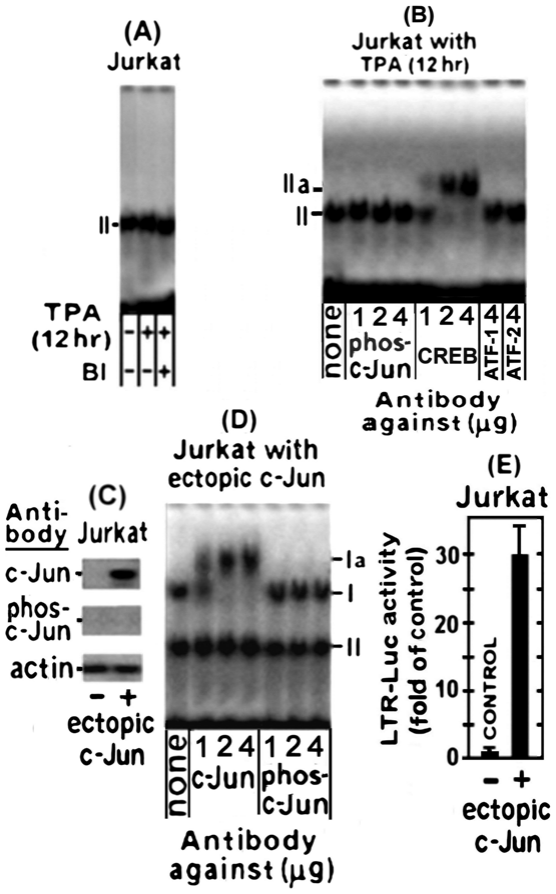
Binding of Jurkat's nuclear proteins to TRE probe. (A) Jurkat cells were treated with TPA ± BI for 12 hr and then their nuclear extracts were analyzed by EMSA for binding to the 3′-biotin-labeled TRE probe. (B) The protein that forms band II was determined by supershift analysis with the indicated amounts of the specified antibodies. (C) Jurkat cells were transfected with ectopic c-Jun expressing plasmid, whereas non-transfected cells served as control. Their whole cell extracts were analyzed at 24 hr after transfection by Western blot with the indicated antibodies for the level of non-phosphorylatedc-Jun (designated as c-Jun-top panel) and phosphorylated c-Jun (designated as phos-c-Jun middle panel). Equal protein loading was assessed by processing the blot of with anti actin antibody (bottom panel). (D) Jurkat cells were transfected with ectopic c-Jun and at 24 hr after transfection their nuclear extracts were subjected to supershift analysis with the indicated doses of the specified antibody. (E) Jurkat cell were co-transfected with LTR-Luc and ectopic c-Jun expressing plasmid. Cells without ectopic c-Jun served as control. Calculation of the enzymatic activities and their presentation were as in Figure 4B.

Next, we examined the TRE oligonucleotide binding by nuclear proteins of TPA-treated Jurkat cells. This was done by exposing the cells to TPA for 12 hr, which was their peak timing of the phospho-c-Jun elevation (see Figure 5A). Figure 6A shows that the nuclear proteins of these cells formed only one band which was not affected by TPA or BI. Since all the TPA-activated PKC isoforms of Jurkat cells have been previously shown to be sensitive to the inhibitory effect of BI [47], the present data indicated that none of the TPA activated PKCs of Jurkat cells was involved in the formation of this band. Furthermore, the present experiment suggested that this band was analogous to band II of H9 cells in terms of its electrophoretic migration (compare Figure 6A and 6B to Figure 2) and supershift pattern by the increasing amounts of anti CREB antibody (compare Figure 6B to Figure 2C). It resembled band II of H9 cells also in being unaffected by excessive amounts of the anti phospho-c-Jun, anti ATF-1 and anti ATF-2 antibodies (compare Figure 6B to Figure 2C) nor by TPA or BI (compare Figure 6A with Figure 2D). Moreover, since TPA elevated in these cells only phospho-c-Jun (see Figure 5A), the lack of band I formation by their extracts (Figures 6A and 6B) provided additional evidence that only non-phosphorylated c-Jun could bind to TREs. This conclusion was further substantiated by the following experiment in which TPA-non-treated Jurkat cells were transfected with a c-Jun-expressing plasmid. Similarly to our finding with H9 cells (Figure 4C), the Western blot analysis depicted in Figure 6C illustrates that this construct produced only non-phosphorylated c-Jun in Jurkat cells too. Consequently, the nuclear extract of these c-Jun-transfected Jurkat cells could now form two EMSA bands with the TRE III probe instead off one. Moreover, the new band was identical to band I of H9 cells in its slower electrophoretic migration and its specific supershift by anti c-Jun antibody but not by the anti-phospho-c-Jun antibody (Figure 6D). In addition, Figure 6E shows that this ectopic c-Jun markedly stimulated the LTR-Luc expression in Jurkat cells. Collectively, these data demonstrated that the non-phosphorylated c-Jun, which was encoded by the ectopic c-Jun plasmid, enabled Jurkat cells to form band I by its interaction with the TREs and to display, thereby, the TRE-dependent LTR activation. This evidence excluded the possibility that some other factors, beside the lack of non-phosphorylated c-Jun elevation, might prevent the TRE-dependent LTR activation in these cells in response to TPA treatment.

### Sp1-p53 complex mediates the second LTR activation phase in TPA-treated H9 cells

**Figure 7:**
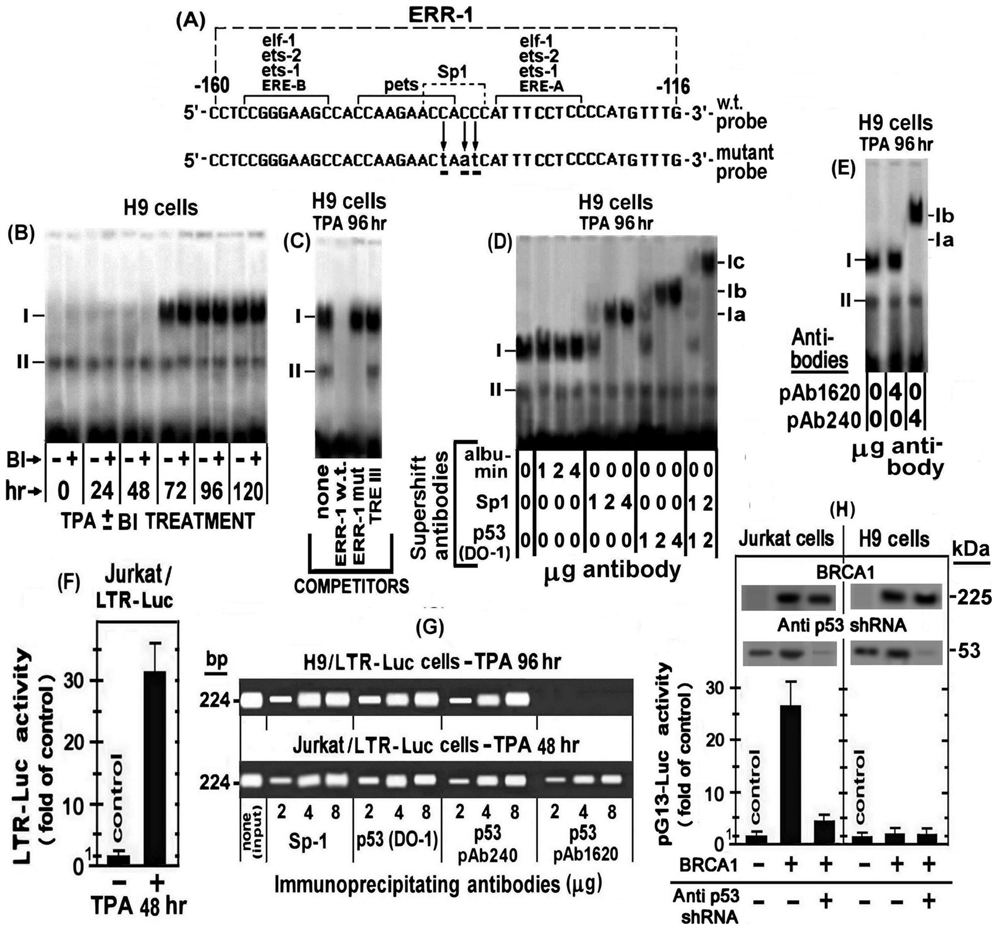
Binding of the Sp1-p53 complex to the Sp1 site of the ERR-1 in TPA ± BI treated H9 cells. (A) Schematic illustration of the ERR-1 probe sequene: The upper probe carries the wild type sequence whereas the lower probe is mutated by 3 substituted nucleotides within the Sp1 binding site depicted in small underlined letters. (B) H9 cells were treated with TPA ± BI for the indicated times and aliquots of their nuclear extracts were tested for binding to 3′ biotin-labeled w.t. ERR-1 probe by EMSA. (C) The specificity of this protein binding to the ERR-1 probe was determined in nuclear extract of cells treated with TPA for 96 hr (i.e. at the second phase of the LTR activation). This was done by competition with 50 molar excess of unlabeled oligonucleotide carrying the w.t. or the mutated ERR-1 sequence. For additional control we employed the same excess of the oligonucleotide carrying the TRE III sequence (shown in “Materials and Methods” and Figure 2A) as an irrelevant competitor. (D) The proteins bound to the ERR-1 probe, were identified by supershift analysis of the nuclear extract derived from H9 cells treated with TPA for 96 hr using 1, 2 and 4 µg ofthe following antibodies: anti albumin (as an irrelevant negative control), or with anti Sp1 and the DO-1 anti p53 (which detect both the w,t. and mutant p53 proteins). A combination of anti Sp1 and anti p53 antibodies (1 and 2 µg of each of them) was employed in the last 2 lanes at the right side of the blot, whereas no antibody was employed in the control cells presented in the first lane at the left side of the blot. (E) To determine whether the Sp1-p53 complex that bound to the ERR-1 probe contained w.t. or mutant p53 protein, a similar supershift analysis was performed with pAb1620 antibody which identified only w.t. p53 and pAb240 antibody that identified only mutant p53. (F) To demonstrate the intracellular recruitment of these nuclear proteins to the ERR-1 site in LTR integrated within the cellular genome, we carried out chromatin immunoprecipitation (ChIP) analysis in the H9 cells stably transfected with LTR-Luc (H9/LTR-Luc) which were treated with TPA for 96 hr. The immunoprecipitation was performed with 2, 4 and 8 µg of each of the indicated antibodies. (G) Jurkat/LTR-Luc cells were tested for activation of their integrated LTR-Luc by 48 hr-TPA-treatment. Untreated Jurkat/LTR-Luc cells served as control. (H) Jurkat (left panel) and H9 (right panel) cells were co-transfected with the pG13-Luc reporter and BRCA1-expressing plasmid in the absence and presence of anti p53-shRNA. Cells without BRCA1 expressing plasmid served as basal control.

In a previous study we have shown that the PKC-antagonized LTR activation in Jurkat cells is mediated by binding of an Sp1-p53 complex to the Sp1 site located within the ERR-1 of the LTR [49]. Subsequently we have noted that the second phase of the LTR activation in TPA-treated H9 cells is exerted through the same site in ERR-1 [47]. Therefore, it was of interest to find out whether this second LTR activation phase was mediated by a similar interaction of Sp1-p53 complex with ERR-1. We addressed this question by analyzing the binding of nuclear proteins of the TPA±BI-treated H9 cells to the 3′-biotin-labeled w.t. ERR-1 probe described in “Materials and Methods”. The EMSA results depicted in Figure 7 B illustrate that during the first 48 hr of the TPA treatment only one band was noted at position II, whereas at later stages of this treatment (i.e. during the second phase of the LTR activation), an additional slower and much thicker band was detected at position I. The specificity of these binding was confirmed by competition analysis of the extract derived from cells treated with TPA for 96 hr, which was arbitrarily chosen to represent the second LTR activation phase. Figure 7 C shows that both bands were competed out by a 50 fold excess of unlabeled w.t. ERR-1 oligonucleotide, while the same excess of the mutated ERR-1 oligonucleotide which carried 3 nucleotide substitutions within its Sp1 site (see the schematic illustration in Figure 7A), eliminated the lower band (band II) but not the upper one (band I). As shown in Figure 7A, ERR-1 includes also binding sites for several additional factors. It is therefore, possible that one or more of these sight might, perhaps, account for the formation of band II regardless of the Sp1 site structure [74]–[77]. However, since this band was unaffected by TPA, it seemed to be irrelevant to the TPA-induced LTR activation and therefore, we did not try to identify its protein composition. Furthermore, the unlabeled TRE III oligonucleotide (shown in Figure 2A), which we employed as an irrelevant competitor, had no effect on either of these bands, thus further substantiating the binding specificity of these bands. Interestingly, these two bands appeared to be analogous to bands I and II that we have previously noted in the TPA-treated Jurkat cells [49]. Moreover, in both cell types band I was detected only after a substantial delay of 12–24 hr in Jurkat [49] and 48–72 hr in H9 (Figure 7A) cells. Of note, however, BI diminished this delay in Jurkat [49], but not in H9 cells (Figure 7B). This finding is consistent with our previous notion that the delay in the onset of the second LTR activation phase in H9 cells is caused by the BI-resistant PKCδ [47].

Figure 7D illustrates a supershift analyses of the nuclear extract derived from the H9 cells treated with TPA for 96 hr, i.e. during the second LTR activation phase. This analysis revealed that increasing amounts of anti Sp1 antibody supershifted band I to position Ia in a dose-dependent manner, whereas similar doses of the DO-1 antibody, which recognized both w.t. and mutant p53 proteins, displayed a stronger supershift that moved band I to position Ib. These different supershifts could be interpreted as indicating that band I represented two separate DNA-protein complexes, one with Sp1 alone and the other with p53 alone, that happened to display a similar electrophoretic mobility but they were differently supershifted by their respective antibodies which formed, perhaps, complexes with different structural configuration. However, this possibility was ruled out by our finding that when the anti Sp1 and the DO-1 antibodies were applied together band I was not splitted to the distinct positions Ia and Ib, but was rather supershifted further as a single band to position Ic. No supershift was imposed by the irrelevant anti albumin antibody (Figure 7D), thus verifying the specificity of this assay. These supershift data closely resembled our previous observation with nuclear extract derived from Jurkat cells after 48 hr of TPA treatment [49]. Of note, however, Figure 7E shows that pAb240 antibody, which recognizes only mutant p53, displayed the same supershift of band I as the DO-1 antibody, whereas the pAb1620 antibody, which recognizes only w.t. p53, did not impose any supershift on band I. These data are contrasting our previous finding with Jurkat cells which have demonstrated that both pAb240 and pAb1620 antibodies formed two new different supershifted bands while leaving a portion of the original band I unshifted [49]. Therefore, we re-assessed our present observation by subjecting H9 and Jurkat cells to chromatin immunoprecipitation (ChIP) assay that was performed with their subclones stably transfected with the LTR-Luc construct (H9/LTR-Luc). The integrated LTR-Luc was effectively stimulated by TPA in both H9 (see Figures 3D) and Jurkat (see Figures 7F) cells. The upper row of Figure 7G shows that anti Sp1 and the DO-1 anti p53 antibodies pulled down comparable amounts of the real-time PCR-amplified DNA fragments obtained from the chromatin of H9/LTR-Luc cells treated with TPA for 96 hr. Since these fragments do not contain specific sites for direct p53 binding [74]–[77], the ability of the DO-1 anti p53 antibody to pull them down indicates that p53 was recruited to ERR-1 through its physical protein-protein association with the Sp1 protein. This row of Figure 7G shows also that the pAb240 antibody, which recognizes only mutant p53, could precipitate ERR-1 fragments, whereas the pAb1620 antibody, which recognizes only w.t. p53, could not. By contrast, the lower row of Figure 7G demonstrates that all the three anti p53 antibodies pulled down comparable amounts of the fragmented chromatin from the TPA-treated Jurkat cells as the anti Sp1 antibody. This discrepancy can be explained by our previously reported evidence that Jurkat cells contain both w.t. and mutant forms of p53 [49], whereas H9 cells contain only mutant p53 [23]. To further substantiate this notion we examined these two cell lines for endogenous w.t. p53 specific transcriptional activity by using a reporter driven by a minimal promoter attached to 13 copies of the w.t. p53 specific responsive site (pG13) as target for this activity. The breast cancer sensitivity BRCA1 protein has been reported to act as transcriptional coactivator of the w.t. p53 protein by physically interacting with this protein and enhancing, thereby, its transcriptional function [78], [79]. Based on this information Jurkat and H9 cells were examined for BRCA1-inducibale endogenous w.t. p53 transcriptional activity by co-transfecting the cells with the pG13-Luc reporter and BRCA1-expressing plasmid. The left panel of Figure 7H shows that this co-transfection strongly activated the pG13-Luc reporter in Jurkat cells. Moreover, this activation was suppressed by anti p53 specific shRNA, thus confirming that it was mediated by an endogenous w.t. p53 existing in these cells. In contrast, no such pG13-Luc activation was detected in H9 cells (Figure 7H, right panel). The Western blots inserted in both panels of Figure 7H illustrate that the BRCA1 and the anti p53 shRNA plasmids displayed equally high expression in both cells. This finding, together with our earlier reported data [23], [49], confirmed that unlike our Jurkat cells, the H9 cells employed in the present study, contain only mutant p53. This mutant is evidently deficient of the w.t. p53 specific transcriptional activity but is still able to interact with certain other factors and to affect their functions.

### Phospho-c-Jun associates with the Sp1-p53 complex and blocks its binding to ERR-1

**Figure 8:**
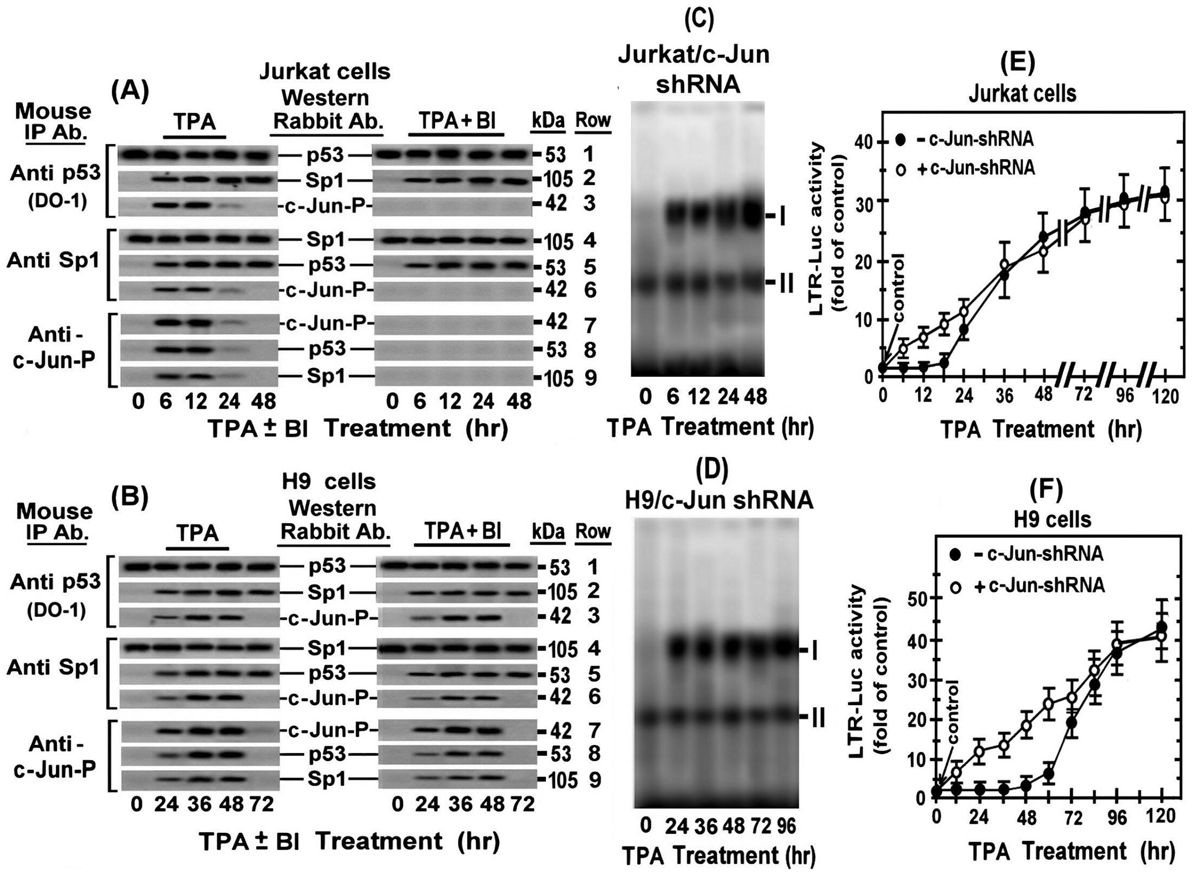
Reciprocal co-immunoprecipitation analysis of phospho c-Jun binding to the Sp1-p53 complex and evidence for its involvement in the delay of the PKC-antagonized LTR activation. Jurkat (A) and H9 (B) cells were treated with TPA for the indicated times in absence (left panels) or presence (right panels) of BI. Aliquots of their nuclear extracts were immunoprecipitated with mouse antibodies (Mouse IP Ab) against p53 (DO-1) (rows 1, 2, 3), Sp1 (rows 4, 5, 6) and phospho-c-Jun (rows 7, 8, 9) as detailed in “Materials and Methods”. The co-immunoprecipitated proteins were dissociated and identified by Western blot analysis with the respective rabbit antibodies (Western Rabbit Ab). To determine whether the phospho-c-Jun binding to the Sp1-p53 complex accounted for the delay of the binding of this complex to ERR-1, c-Jun was knockdown by shRNA in Jurkat (panel C; Jurkat/c-Jun shRNA) and H9 (panel D; H9/c-Jun shRNA) cells. These cells were treated with TPA for the indicated times and their nuclear extracts were examined by EMSA for binding to the 3′-biotin-labeled ERR-1 probe as described in “Materials and Methods). To explore whether the phospho-c-Jun binding to the Sp1-p53 complex accounted for the delay in the onset of the PKC-antagonized LTR activation by TPA, the Jurkat/c-Jun shRNA cells (panel E) were treated with TPA whereas H9/c-Jun shRNA were pretreated with TPA+BI. Cells without the anti c-Jun served as control. At the indicated times of these treatments the cells were transfected with LTR-Luc. The enzymatic activity was measured at 24 hr post-transfection and plotted as describe in Figure 4B.

The time-course of the phospho-c-Jun elevation in the TPA-treated H9 (Figure 1) and Jurkat (Figure 3) cells correlates the delayed binding of the Sp1-p53 to ERR-1 in both H9 (see Figure 7B ) and Jurkat (see ref. [49]) cells. This correlation pointed to the possibility that this delay was imposed by physical interaction of the elevated phospho-c-Jun with the Sp1-p53 complex. We search for such physical interaction by reciprocal co-immunoprecipitation analyses of the nuclear extracts of these cells at the indicated time-points of their TPA treatment. We found that the immunoprecipitates pulled down by the DO-1 anti p53 antibody from the nuclear extracts of the Jurkat (Figure 8A, left panels, rows 1 and 2) and H9 (Figure 8B, left panels, rows 1 and 2) included the Sp1 protein during their entire exposure to TPA. Conversely, the precipitates that were pulled down by anti Sp1 antibody included p53 protein (Figure 8A, left panels, rows 4 and 5 for Jurkat cells and Figure 8B, left panels, rows 4 and 5 for H9 cells). On the other hand, the precipitates that were pulled down by anti p53 or anti Sp1 antibody included phospho-c-Jun (c-Jun-P) only during its elevation time. i.e. 6–12 hr of the TPA treatment in Jurkat cells (Figure 8A, left panels, rows 3 and 6) and 24–48 hr of the TPA treatment in H9 cells (Figure 8B, left panels, rows 3 and 6). Furthermore, the precipitates that were pulled down by the anti c-Jun-P contained all the three protein components in both cells, but only during the phospho-c-Jun elevation time (see Figure 8A left panels for Jurkat cells and Figure 8B left panels for H9 cells). The results depicted in the right panels show that BI did not affect the association between p53 and Sp1 (Figure 8A, right panels, rows 1, 2, 4 and 5 for Jurkat cells and Figure 8B, right panels, rows 1, 2, 4 and 5 for H9 cells). BI abolished, however, the association of c-Jun-P to the Sp1-p53 complex in Jurkat (Figure 8A, right panels, rows 3, 6, 7, 8 and 9), most likely by inhibiting the PKCα and PKCε-mediated phospho-c-Jun elevation in these cells (see Figure 5A, rows 4 and 5). In contrast, BI did not interfere with this association in H9 cells (Figure 8B, right panels, rows 3, 6, 7, 8 and 9), since it could not avoid the phospho-c-Jun elevation by the BI-resistant PKCδ (see Figure 1C, rows 4 and 5). Figures 8A and 8B show also that no association between Sp1 and p53 occurred in the TPA-un-treated Jurkat and H9 cells. In summary, these data together indicated that TPA triggered the association between Sp1 and p53 in both cell lines through a mechanism that was neither dependent on, nor interfered by any of the TPA-activated BI-sensitive PKCs. The possible involvement of theBI-resistant PKCδ in this association within H9 cells was also excluded by our previous observation that silencing PKCδ by specific shRNA did not abort the second phase of the LTR activation in these cells [47].

Notably the binding of phospho-c-Jun to Sp1-p53 coincided with the delay in the Sp1-p53 binding to ERR-1for more than 48 hr of the TPA treatment in H9 (see the above Figure 7B) and for more than 12 hr in Jurkat cells (see ref. [49]). To explore whether the phospho-c-Jun binding to Sp1-p53 caused these delays, we re-assessed the binding of this complex to ERR-1 in the two cells types stably transfected anti c-Jun shRNA. Figures 8C (Jurkat/anti c-Jun shRNA) and 8D (H9/anti c-Jun shRNA) show that knockdown of c-Jun totally alleviated this delay in both cells by permitting this binding immediately after the beginning of the TPA treatment.

Finally, to re-assure that the transient elevation of phospho-c-Jun accounted for the delay in the onset of the PKC-antagonized LTR activation by TPA, we examined the effect of c-Jun knockdown with specific shRNA on this delay. Figure 8E shows that c-Jun silencing diminished this delay in Jurkat cells. For examining the effect of c-Jun silencing in H9 cells, the TPA treatment had to be carried out in the presence of BI in order to avoid the first phase of the LTR activation, since otherwise this first phase would mask any effect on the early steps of the second phase. Figure 8F shows that under such conditions the second phase started, indeed, with no delay.

### Schematic summary of the PKC/c-Jun LTR activation pathways in the TPA-treated H9 and Jurkat Cells

**Figure 9:**
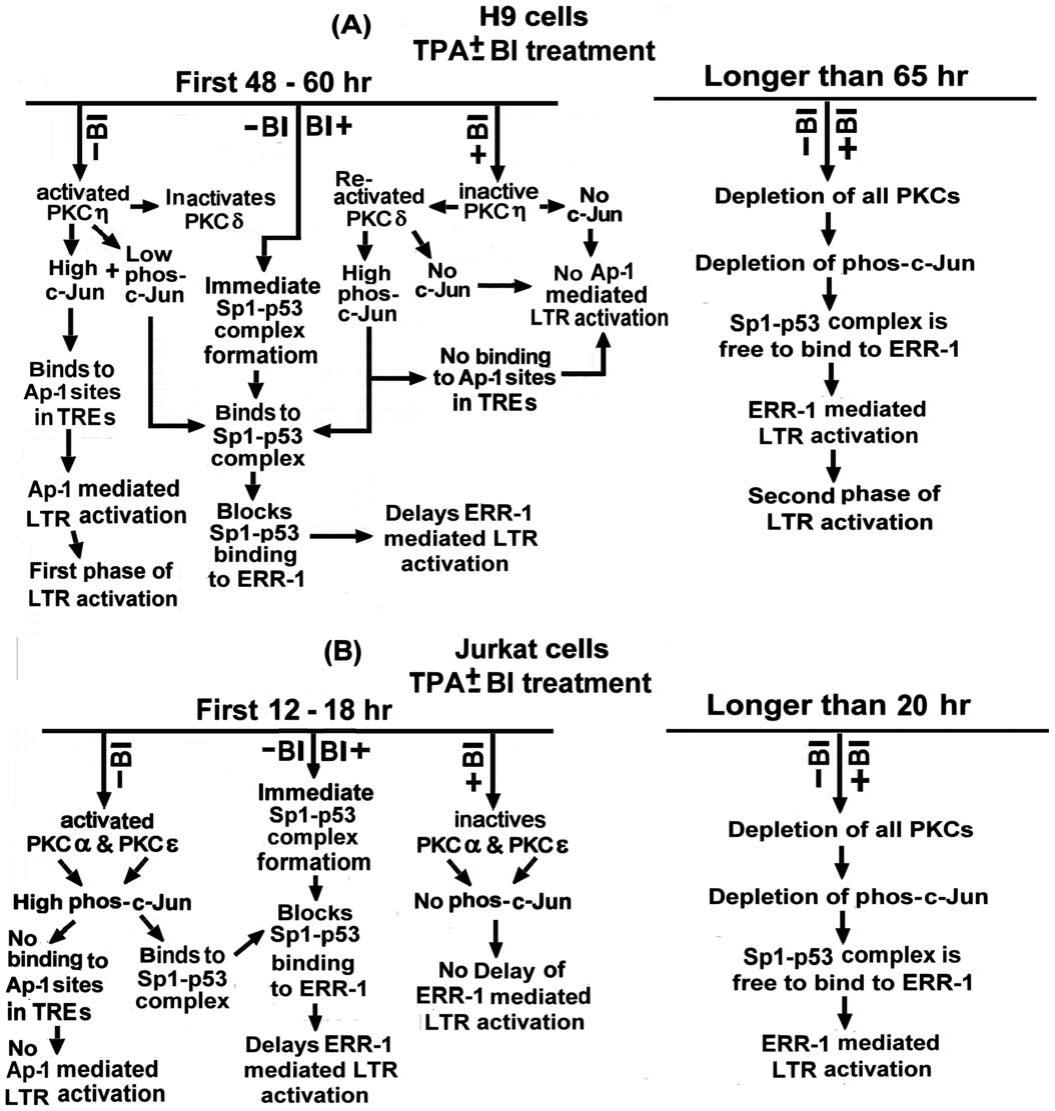
Schematic summary of the TPA-induced LTR activation pathways in (A) H9 and (B) Jurkat cells.

Figure 9 schematically summarizes the different roles of the various TPA-activated PKC isoforms in the HTLV-1 LTR activation in H9 (panel A) and Jurkat (panel B) cells and the functions of the PKC-elevated phosphorylated and non-phosphorylated c-Jun in the different downstream mechanisms of the LTR activation.

## Discussion

Our earlier search for environmental factors that might be involved in reactivation of the dormant virus in HTLV-1 asymptomatic carriers has revealed that HTLV-l LTR can be activated in various human T-cell lines and primary T-lymphocytes by DNA-damaging and other stress-inducing agents in absence of Tax [41]. Of these agents we have focused mainly on the LTR activation by TPA [41], [47]–[49] for the reasons discussed in the “Introduction” section.

We have recently noted that although TPA activates in Jurkat and H9 cells the same set of PKC isoforms (α, β1, β2, δ, ε, η), their functional activities and the rate of their TPA-mediated downregulation varied between these two cell types. For instance, PKCη and PKCη persist in the TPA-treated H9 cells much longer (more than 48 hr) than in Jurkat cells (about 12 hr only). Moreover, we have noted that in H9 cells PKCη activity suppresses the activities of the other isoforms, whereas in Jurkat cells PKCη activity is suppressed by PKCα and PKCε [47]. Along with these differences, we have discovered two distinct mechanisms of TPA-induced LTR activation in these cells. One of them is strictly dependent on PKCη and transiently operates only in H9 until this isoform is depleted from the cells. This mechanism does not operate in Jurkat cells since, as noted above, PKCη activity is suppressed in these cells by PKCα and PKCε [47]. The other mechanism operates in both cells. It is rather antagonized by specific PKC isoforms and, therefore, its onset is delayed until these particular PKCs are depleted from the cell due to their downregulation by the extended exposure to TPA. In H9 cells this mechanism is antagonized by PKCδ [47], whereas in Jurkat cells it is antagonized by a joint action of PKCα and PKCε [41], [47].

In the present study we observed that PKCη induced the first LTR activation phase in H9 cells by transiently increasing the level of c-Jun. A small portion of the elevated c-Jun was phosphorylated at serine residues 63 and 73, whereas its the vast majority was non-phosphorylated. The non-phosphorylated c-Jun proved to induce this LTR activation by its direct binding to the three 21 bp TRE repeats. No such PKC-dependent LTR activation was observed in Jurkat cells, since PKCα and PKCε suppressed the activity of PKCη in these cells [47], while elevating only phospho-c-Jun, which proved to be incapable of binding to the TRE repeats.

In exploring the mechanism of the PKC-antagonized mode of the LTR activation, we have found in our previous studies that in Jurkat cells TPA activates the LTR by inducing the formation of an Sp1-p53 complex which binds to an Sp1 site residing within the ERR-1 domain of the LTR [41], [49]. These studies have also shown that the Sp1-p53 binding to ERR-1 is interfered in these cells by PKCα and PKCε and that this interference can be alleviated by BI [41], [49]. In the present study we found that the phospho-c-Jun, which was cooperatively elevated in these cells by PKCα and PKCε physically interacted with the Sp1-p53 complex and blocked its binding to ERR-1. Consequently, the onset of the LTR activation was delayed for 12–18 hr, i.e. until these two PKCs were depleted from the cells by their downregulation. Notably, the PKC-antagonized LTR activation in H9 cells was delayed for much longer time (48–60 hr) but, in contrast to Jurkat cells, it was unaffected by BI in these H9 cells [47]. In addition, we found here that the BI-abolished inhibition of the Sp1-p53 binding to ERR-1 in Jurkat cells was exerted by blocking the PKCα/PKCε-mediated phospho-c-Jun elevation. The failure of this inhibitor to do so in H9 cells proved to result from the phospho-c-Jun that was elevated in these cells by their BI-resistant PKCδ, which was released from its BI-inhibited PKCη-mediated suppression. TPA and other PKC activators are known for a long time as activators of c-Jun expression and functions [65]. The N-terminal region of the c-Jun protein includes a binding site for the c-Jun N-terminal kinases JNK1, JNK2 and JNK3 (JNKs). It contains also phosphorylation sites and transactivation domain [80]. Under certain conditions the c-Jun N-terminal phosphorylation by JNKs at seine residues 63 and 73 and theronine residues 91 and 93, stimulates the c-Jun transcriptional activity, whereas in some other conditions the phosphorylation of serine 63 and 73 increases c-Jun capacity to physically interact with other binding partners [80], [81]. Notably the phospho-c-Jun detected in our experiments, was proven to be phosphorylated at serine 63 and 73, by showing that it was recognized by an antibody that specifically identifies these phosphorylated residues (see Materials and Methods). It is, therefore, reasonable to assume that this specific phosphorylation accounted for its physical association with the Sp1-p53 complex observed in our experiments. Moreover, several recent reports, which have shown that TPA can induce JNK-mediated c-Jun phosphorylation in certain cells [82]–[85]. On this ground, we speculate that the TPA-activated PKC isoforms, which elevated the phospho-c-Jun level in our present study, exert this effect indirectly through specific JNK activation.

Interestingly, while the binding of the Sp1-p53 to ERR-1 has been noted in our previous studies to be interfered by PKC [41], no effect of PKC was detected in the present study on the formation of this complex in H9 cells, which occurred in these cells shortly after TPA addition to the cells. Based on these findings we hypothesize that TPA triggers the apoptotic cascade by inducing PKC-independent DNA-damaging oxidative burst [86] and that certain, undefined yet, factors of the early stage of the apoptotic cascade might induce this rapid Sp1-p53 complex formation.

EMSA analysis of the Sp1-p53 binding to ERR-1 revealed that the DO1 antibody, which recognized both w.t. and mutant p53 proteins, displayed a stronger supershift of the Sp1-p53 related band than the anti Sp1 antibody. This could argue that band I consisted of two complexes, that happened to display similar electrophoretic mobility, but could be separately supershifted by their specific antibodies. This argument was declined by our observation with extracts of Jurkat cells that employing the anti Sp1 and anti p53 antibodies together shifted the original band I as a single band to a higher position III. If band I consisted, indeed, of two separate complexes, then the shift with the two antibodies together should have splitted it into two separate bands. In addition, we have found in our previous study [49] that the pAb1620 antibody, which recognizes only w.t. p53, and the pAb240 antibody, which recognizes only mutant p53, formed two new different supershifted bands while leaving a portion of the original band unshifted [49]. Together with this latter finding, it appears that p53 exists in this complex at a two fold higher molar quantity than Sp1, which in the case of Jurkat cells, it includes both the w.t. and the mutant p53 forms. Therefore, higher amount of the DO1 anti p53 than the anti Sp1 antibody can bind to the band I complex. Furthermore, in another study with Jurkat cells we have observed that the Sp1-p53 complex binds also to an Sp1 recognition site residing in the p21Waf-1 promoter and presents similar EMSA and supershift patterns [87]. Thus, while several laboratories claim that their Jurkat cells are p53-null [88], [89], our findings in these studies indicate that our Jurkat cells contain comparable levels of both w.t. and mutant p53 proteins. Moreover, in the present study we demonstrated that Jurkat cells contained p53 protein that displayed w.t. p53 specific transcriptional activity. Similar p53 status was observed also by Gualberto et al in their Jurkat cells [90], [91]. In contrast, analyses, performed in the present study with H9 cells, revealed that these cells contain only mutant p53. This notion is compatible with earlier reported immunostaining [92] and immunoprecipitation [49], [92] analyses which could detect in H9 cells only mutant p53. However, it remains unclear from these data whether only complexes with mutant p53 molecules, which are formed in both Jurkat and H9 cells, are effective in activating the LTR or whether those with w.t. p53 formed in Jurkat cells are effective as well.

Finally, it is important to note that p53 is one of the most commonly mutated genes in human cancers [93], [94]. Many different mutations have been identified in p53 of tumor cells. Most of these mutations abrogate its specific affinity towards the w.t. p53 DNA responsive element, while others confer upon it new oncogenic properties [95] or affect its protein conformation [96]. However, very little is known about the linkage between particular p53 mutations to specific clinical outcomes [93], [94].

### Conclusions

Our results raise the question as to which of the two mechanisms discussed above operates in the infected T-cells of HTLV-1 carriers. We like to emphasize that in addressing this question it should be kept in mind that the individual infected T-cells of the HTLV-1 carriers are likely heterogenic and vary in several aspects. Of particular relevance to the viral reactivation issue is the variation in the level of the viral gene expression between different individual infected T-cells, which ranges from high to very low expression. The level of the viral gene expression in each individual infected cell is likely determined by the integration site of the proviral DNA in the host cell genome. Integration within condensed DNA regions permits lower viral gene expression than in relaxed DNA regions. Cells with high viral gene expression are believed to be eliminated by the host immune surveillance [14], [97]. Therefore, it should not be surprising if PKC-activating physiological or pathological conditions may reactivate the latent virus in different infected individual cells by different mechanisms. Furthermore, we are currently investigating the mechanisms of LTR activation by other PKC-unrelated stress-inducing agents that might differ from those associated with PKC. Since blocking the latent virus reactivation is highly important for preventing the leukemic process in HTLV-1 carries, it is essential to explore all possible reactivation mechanisms in order to develop specific drugs against each of them. These drugs should be prescribed as cocktails that HTLV-1 carriers will have to take for their entire life as is currently done, for example, with HIV carriers and certain other clinical conditions.

## Materials and Methods

### Plasmids and transfection

The reporter plasmid expressing the firefly luciferase through HTLV-1 LTR (LTR-Luc) was provided by Susan J. Marriott (Baylor College of Medicine, Houston, TX). The plasmid expressing w.t. Tax through the CMV promoter, was provided by Francoise Bex (Laboratore de Microbiologie, Universite Libre de Bruxelles, B-1070, Brussels, Belgium). The plasmid pG13-Luc, expressing luciferase through a minimal promoter containing 13 copies of the w.t. p53-consensus binding sequence, was obtained from Moshe Oren (Weizman Institute of Science, Rehovot, Israel). The BRCA1-expressing plasmid [60] was from Haim Werner (Department of Clinical Biochemistry, Sacler School of Medicine, Tel Aviv University, Tel Aviv, 69978, Israel). The pZL plasmids expressing constitutively active PKC isoform α, β1, β2, γ, δ, ε and η [61] were provided by Etta Livne (Faculty of Health Sciences, Ben-Gurion University of the Negev, Beer-Sheva, Israel) and described in our previous article [47]. The plasmid expressing the Renilla luciferase via the enhancerless promoter pRL-null (pRL-renilla) was purchased from Promega Corporation (Madison WI, USA). Sets of five plasmids with Puromycin selection marker, each expressing a different shRNA clone for each of the studied target genes, were purchased from Sigma-Aldrich Corporation. To choose the most appropriate shRNA clones for our experiments their silencing efficiency and specificity were tested by Western analysis of their target proteins with comparison to several randomly selected unrelated proteins (not shown). Plasmids were transfected at the indicated combinations by the jetPRIM™ kit (Polyplus transfection, www.polyplus-transfection.com) according to the manufacturer's instructions with adjustments to a total 5 µg DNA per transfection. The transfection efficiency, tested with GFP-expressing plasmid, was found by FACS analysis to range in our employed cells between 70 to 80% (not shown). Each transfection mixture included the pRL-renilla plasmid as an internal control for variation in transfection efficiency. The enzymatic activities were measured at 24 hr post-transfection and the Luc activity was normalized to that of renilla and presented as fold of the relevant control. The plotted results represent the average of triplicate transfections ± SE.

### Cells and culture conditions

Most experiments of this study were carried out with Jurkat and H9 T-cells lines (kindly provided by Prof. Irvin S.Y Chen, Center for HIV and digestive diseases, UCLA AIDS Institute, Los Angeles, California, USA). In addition, we generated from these cells sub-clones stably expressing shRNAs which were designated after their target genes as follows: Jurkat/PKCα-shRNA, Jurkat/PKCε-shRNA, Jurkat/p53-shRNA, Jurkat/c-Jun-shRNA, H9/PKCδ-shRNA, H9/PKCη-shRNA, H9/p53-shRNA and H9/c-Jun-shRNA, We also generated Jurkat and H9 cells stably transfected with HTLV-1 LTR-Luc. These sub-clones were designated as Jurkat/LTR-Luc and H9/LTR-Luc. These latter two clones were validated by checking the activation of their Luc expression by transient transfection with the Tax expressing plasmid (not shown). The cells were grown in RPMI-1640 supplemented with 10% fetal calf serum and antibiotics.

For long-term maintaining of stably transfected clones, they were grown in the presence of the selective antibiotics to avoiding overgrowth of cells that have lost the transfected construct. However when these cells were prepared for experiments they were grown for 1–2 days without the antibiotics to avoid their possible side effects.

In experiments lasting more than 48 hr, the cells were sub-cultured every two days into fresh medium containing the same experimental ingredients and keeping approximately the same cell density in order to avoid lowering of the medium ingredients and to minimize cell death due to culture aging. Cell viability was checked by the trypan blue expulsion analysis.

### Antibodies

The DO-1 mouse monoclonal antibody which recognizes both the w.t. and mutant p53 configurations and the mouse anti actin antibody, were purchased from Calbiochem and ICN respectively. Mouse polyclonal antibodies against Sp1, c-Jun, CREB, ATF-1, ATF-2 and albumin, goat antibody specifically detecting c-Jun phosphorylated at serine residues 63 and 73, (referred to as anti phospho-c-Jun antibody), rabbit polyclonal antibodies against p53, mouse anti p53 monoclonal antibody pAb240 (recognizing only mutant forms of p53) and pAb1620 (recognizing only w.t. p53), rabbit anti mouse-IgG, mouse anti rabbit-IgG and anti goat-IgG antibodies, were all purchased from Santa Cruz Biotechnology Inc, Santa Cruz, CA, USA.

### TPA treatment in absence and presence the PKC inhibitor bisindolylmaleimide-I (BI)

The cells were exposed to 50 nM TPA (Sigma-Aldrich Corporation) for the indicated times. Where specified, 2 µM of the PKC inhibitor BI [41], [59] was added one hour before TPA to ensure its presence within the cells before PKC activation.

### Cell fractionation, co-immunoprecipitation and Western blot analyses

Whole cell lysates and subcellular fractions were prepared by NucBuster Kit (Calbiochem, Cat. No. 71183-3), according to the Kit's protocol.

For co-immunoprecipitation assays aliquots of the nuclear extracts (200 µg protein) were immunoprecipitated with the specified mouse antibodies (Mouse IP Ab). The precipitates were collected with protein-A/protein-G bound Sepharose beads, washed twice to remove non-specific proteins. Then they were dissociated and analyzed by Western blot for the co-precipitated proteins with the respective rabbit antibodies as previously described [41]. The specificity of the assay was verified by subjecting the extracts to co-immunoprecipitation with non-specific mouse IgG, For direct Western analyses aliquots of the tested extracts (80 µg protein) were analyzed with the respective antibodies as described elsewhere [48]. Equal loading of samples was assessed by stripping the blot from the first antibodies and re-processing it with anti-actin antibody.

### Electrophoretic mobility shift and supershift assay

Aliquots of 6 µg nuclear proteins of the tested cells were examined for binding to 3′-biotin-labeled double stranded oligonucleotides carrying the sequence of the 21 bp TRE III (its upper strand is 5′-CAGGCGT**TGACGACA**ACCCCT-3′. The bold letters represent a core octanucleotide sequence which is an imperfect match of the cyclic AMP responsive element, CRE, that partially overlaps the phorbol ester/AP-1 responsive element [62]) or of the Ets responsive region 1 (ERR-1) of the LTR (its upper strand is 5′-CCTCCGGGAAGCCACCAAGA**ACCACCC**ATTTCCTCCCCATGTTTG-3′. The bold letters represent an Sp1 binding site) by electrophoretic mobility shift assay (EMSA) using the LightShift**®** Chemiluminescent EMSA kit (PIERCE, Rockford, IL) as instructed by the supplier. The binding specificity was assessed by competition with 50 fold molar excess of unlabeled oligonucleotide of the relevant probe versus its mutated oligonucleotide carrying the following nucleotide substitutions (marked by underlined bold small letters): In TRE III: 5′-CAGGCGT**TGAtGACA**ACCCCT-3′ and ERR-1: 5′-CCTCCGGGAAGCCACCAAGA**ACtAatC**ATTTCCTCCCCATGTTTG-3′, The bound proteins were identified by supershift analysis with the respective antibodies according to the kit's protocol. To increase the accuracy and reliability of the supershift data we applied increasing amounts (1, 2 and 4 µg) of each of the employed antibodies.

### DNA-protein pull-down assay

Nuclear extracts (200 µg protein) of the tested H9 and Jurkat cells were mixed with 1 µg or 6 µg of the above mentioned 3′-biotin-labeled TRE III or ERR-1 oligonucleotide probes and 10 µg sheared salmon sperm DNA in pull-down buffer (10 mM HEPES, pH 7.9, 25 mM KCl, 500 µM DTT, 2.5% glycerol and protease inhibitor cocktail) and incubated in ice for 90 min. Then Streptavidin-linked agarose beads (70% slurry) were added and the mixtures were gently rotated overnight at 4°C. The beads were then pelleted and washed with cold pulled-down buffer. The bound proteins were released from the beads by heating at 95°C for 5 min, separated by 9% SDS polyacrylamide gel electrophoresis (PAGE) and identified by Western blot analysis with the indicated antibodies.

### Chromatin immunoprecipitation assay (ChIP)

HTLV-1 LTR-Luc stably-transfected Jurkat (Jurkat/LTR-Luc) and H9 (H9/LTR-Luc) cells (2×10^7^) were treated with TPA for the indicated time and their chromatin was cross-linked by adding formaldehyde (1% final concentration) to the cultures for 10 min at room temperature. The formaldehyde was quenched by glycine (125 mM final concentration) with shaking for 5 min. Then the cells were washed 3 times with ice-cold PBS and suspended in 750 µl lysis buffer (1% SDS, 10 µM EDTA, 50 µM Tris-HCl and protease inhibitor cocktail). The lysates were sonicated (10×15 sec pulses) on ice to generate 400–600 bp fragments and centrifuged for 30 sec at 4°C to remove cell debris. The supernatants containing the fragmented chromatin were diluted 10 fold in ChIP buffer (1% Triton X-100, 2 µM EDTA, 150 µM NaCl, 20 µM Tris-HCl and protease inhibitor cocktail), cleared by incubation with protein-A/protein-G bound Sepharose beads for 2 hr at 4°C and the cleared supernatant was splitted into equal aliquots. One aliquot was saved for quantifying the input DNA, whereas the others were immunoprecipitated by incubation with the indicated amounts (2–8 µg) of the tested antibodies for overnight at 4°C. The immunoprecipated chromatin fragments were collected by incubation with the protein-A/protein-G bound Sepharose beads for 1 hr at 4°C, washed 3 times with ChIP buffer. The DNA-protein cross-linking of these and the input aliquots was reversed by incubation in ChIP buffer containing proteinase K for 2 hr at 65°C and then for additional 10 min at 95°C. The free DNA fragments were purified by the QIAquick PCR Purification kit (Qiagen) and amplified by real-time PCR using primers that flanked the segment between nucleotides −321 and −97 in the U3 region of the integrated LTR. This segment included the three 21 bp TRE repeats and the ERR-1 region which is located between TRE-II and TREIII (forward: 5′-TTCCGAGAAACAGAAGTCTG; reverse: 5′-GTGAGGGGTTGTCGTCA (see ref [63], [64]). The PCR DNA products were analyzed by 1.2% agarose gel electrophoresis.
